# A comparison of 4D time-resolved MRA with keyhole and 3D time-of-flight MRA at 3.0 T for the evaluation of cerebral aneurysms

**DOI:** 10.1186/1471-2377-12-50

**Published:** 2012-07-06

**Authors:** Qian Wu, Ming-Hua Li

**Affiliations:** 1 Dept of Radiology, The Sixth Affiliated People’s Hospital; Medical school of Shanghai Jiaotong University, No. 600# Yishan Road, Shanghai, 200233, China

**Keywords:** 4D time-resolved MRA with keyhole, 3D time-of-flight MRA, DSA, Cerebral aneurysm

## Abstract

**Background:**

A subarachnoid hemorrhage (SAH) due to the rupture of a cerebral aneurysm (CA) is a devastating event associated with high rates of mortality. Magnetic resonance angiography (MRA), as a noninvasive technique, is typically used initially. The object of our study is to evaluate the feasibility of 4D time-resolved MRA with keyhole (4D-TRAK) for the diagnostic accuracy and reliability of the detection and characterization of cerebral aneurysms (CAs), with a comparison of 3D time-of-flight MRA (3D-TOF-MRA) by using DSA as a reference.

**Methods:**

3D-TOF-MRA, 4D-TRAK and 3D-DSA were performed sequentially in 52 patients with suspected CAs. 4D-TRAK was acquired using a combination of sensitivity encoding (SENSE) and CE timing robust angiography (CENTRA) k-space sampling techniques at a contrast dose of 10 ml at 3 T. Accuracy, sensitivity, specificity of 4D-TRAK and 3D-TOF-MRA were calculated and compared for the detection of CAs on patient-based and aneurysm-based evaluation using 3D-DSA as a reference.

**Results:**

The overall image quality of 4D-TRAK with a contrast dose of 10 ml was in the diagnostic range but still cannot be compared with that of 3D-TOF-MRA. In 52 patients with suspected CAs, fifty-eight CAs were confirmed on 3D-DSA finally. Fifty-one (with 2 false-positives and 9 false-negatives) and 58 (with 1 false-positive and 1 false-negative) CAs were visualized on 4D-TRAK and 3D-TOF-MRA, respectively. Accuracy, sensitivity and specificity on patient-based evaluation of 4D-TRAK and 3D-TOF-MRA were 92.31%, 93.33%, 85.71% and 98.08%, 100%, 85.71%, respectively, and 74.07%, 75.00%, 66.67% and 96.30%, 95.83%, 100% on aneurysm-based evaluation in patients with multiple CAs, respectively. Subgroup analysis revealed that for 19 very small CAs (maximal diameter <3 mm, measured on 3D-DSA), 9 were missed on 4D-TRAK and 1 on 3D-TOF-MRA (P = 0.008). However, for 39 CAs with maximal diameter ≥ 3 mm, the diagnostic accuracy is equally (39 on 4D-TRAK vs. 39 on 3D-TOF-MRA) (P = 1). In four larger CAs with maximal diameter ≥ 10 mm, 4D-TRAK provided a better characterization of morphology than 3D-TOF-MRA.

**Conclusion:**

4D-TRAK at a lower contrast dose of 10 ml with a combination of SENSE and CENTRA at 3 T could provide similar diagnostic accuracy rate for CAs with maximal diameter ≥ 3 mm, and a better characterization of morphology for larger CAs with maximal diameter ≥ 10 mm compared to 3D-TOF-MRA. However, further study is still needed to improve the “vascular edge” artifact and the compromise in spatial resolution in depiction of CAs with maximal diameter<3 mm.

## Background

A subarachnoid hemorrhage (SAH) due to the rupture of a cerebral aneurysm (CA) is a devastating event associated with high rates of mortality. Digital subtraction angiography (DSA) combined with post-processing techniques remains to be the gold standard for detecting and evaluating CAs [1,2]. However, risks include vascular injury, contrast nephrotoxicity, exposure to ionizing radiation and an overall 0.2% risk of transient and permanent neurological complications [3]. Therefore, as a noninvasive technique, magnetic resonance angiography (MRA) is typically used initially. Although numerous studies have proved that 3D time-of-flight MRA (3D-TOF-MRA) is an effective technique in the detection and characterization of CAs [4,5], disadvantages including long acquisition time, small FOV and flow related artifact remain the problem. The 4D time-resolved MRA with keyhole (4D-TRAK) and a combination of sensitivity encoding (SENSE) and CE timing robust angiography (CENTRA) k-space sampling has demonstrated its wide clinical potential in the characterization of hemodialysis shunts, pulmonary vascular pathologies and cerebral arteriovenous malformations (AVMs) [6-9]. The clinical potential in CAs of this combination of such image acceleration methods includes: 1) better characterization of the morphology of larger CAs better due to its reduced flow related artifact; 2) preoperative assessment of endovascular therapy of CAs by mimicking DSA because of its high spatial and temporal resolution at a larger FOV; 3) fewer motion artifacts as a consequence of the shortened acquisition time for patients with acute aneurysmal SAH. The purpose of our study was to evaluate the feasibility of 4D-TRAK for the diagnostic accuracy and reliability of the detection and characterization of CAs, with a comparison of 3D-TOF-MRA by using DSA as a reference and to discuss their respective advantages and disadvantages.

## Methods

### Study subjects

From June 2008 to February 2010, 52 patients with suspected CAs (20 men, 32 women; age range 31–78 years) were finally enrolled into our study and 6 of them were with acute SAH detected by CT scan. 3D-TOF-MRA, 4D-TRAK and 3D-DSA were performed sequentially to detect the CA occurrence. The study was conducted in accordance with the recommendations of the Declaration of Helsinki, and was approved by the ethics committees in the hospitals. All patients gave written informed consent. Exclusion criteria included age below 18 years, contraindications to MRA examination (e.g. pacemakers, metallic implants), known intolerance to gadolinium contrast agents, with stage 5 CKD (Chronic Kidney Disease, effective glomerular filtration rate<15 ml/min/1.73 m [2]) or on dialysis, no informed consent from patient or qualifying family members, poor neurological condition (GCS < 12), without DSA information acquired contemporaneously or having undergone MRA after a coiling procedure.

### Image acquisition

#### MRA

All MRA examinations were performed on a 3.0 T system (Achieva X-Series, Philips Healthcare, The Netherlands) with a Sense-Head-8 receiver head coil. 3D-TOF-MRA was conducted using a 3D-T1-FFE sequence with TR/TE, 35/7; flip angle, 20°; field-of-view (FOV), 250 × 190 × 108; four slabs (180 slices); slice thickness, 0.8 mm; matrix, 732 × 1024; and acquisition time, 8 min 56 s. We used the 4D-TRAK technique combined with CENTRA k-space sampling and sensitivity encoding (SENSE) to accelerate the image acquisition. 4D-TRAK was acquired using the CENTRA keyhole method with 20% in the sagittal plane. A central space is randomly filled during the whole passage of the contrast bolus over time and the periphery of k-space was collected in the reference dataset at the end of the acquisition. Sensitivity encoding (SENSE) was used with an acceleration factor (AF) of 4 in the phase-encoding direction and an AF of 2 in the slice-encoding direction yielding a total AF of 8. The acquisition parameters of the 4D-TRAK technique were as follows: 3D-T1-GE sequence with TR/TE, 3.1/1.2; flip angle, 25°; FOV, 230 × 190 × 108; four slabs (273 slices); slice thickness, 0.8 mm; matrix, 201 × 320; and acquisition time, 40 s (reference scanning time, 12 s; 2 s per phase for 14 phases in total). The 4D-TRAK sequence was started immediately after the start of the injection. A 10-ml bolus of gadopentetate dimeglumine (Gd-DTPA, Magnevist, Bayer-Schering, Germany) was administered at 3 ml/s and followed by a 20 ml saline injection using a dual-barrel power injection machine at the same rate.

The acquired image data sets were transferred to a workstation (EWS, Philips Medical), where 3D image reconstruction was performed on a 1024 × 1024 matrix with volume rendering (VR) and maximum intensity projection (MIP) using a specialist software package (Volume Inspection, Philips Medical).

#### DSA

For patients with acute SAH, an interventional neuroradiologist performed DSA within 24 hours after MRA examination, and for those with suspected un-ruptured CAs, DSA was performed within 2 weeks after MRA (between 1 h and 14 d post-MRA; median, 10.6 days). Conventional 2D-DSA was performed on a monoplanar unit (Axiom Artis VB22N, Siemens Healthcare, Germany) with a 1024 × 1024 matrix and 17–20 cm FOV. Contrast medium was injected for a total of 10 ml for internal carotid artery (ICA; 4–5 ml/s) and 7 ml for vertebral artery (VA, 2–3 ml/s) respectively. Rotational angiography was performed with an 8-s, 200° rotational run, acquiring 200 images and with the injection of 3–4 ml contrast material per second (total 16–20 ml/artery). 3D images were reconstructed by VR on a workstation with a 128 [3] × 512 [3] matrix (SyngoXWP VA70B, Siemens).

All patients with possible CAs underwent 2D-DSA (including posterior-anterior, lateral and working positions) and 3D-DSA of the affected arteries. 2D-DSA (including posterior-anterior and lateral positions) was performed for the rest of the arteries, and further 3D-DSA would be performed with any positive findings on 2D-DSA. Two experienced observers, who were unaware of all clinical information, identified and evaluated the CAs together.

### Image review

We considered a CA to be a saccular protrusion from the side wall or bifurcation of the cerebral arteries without artery emerging at its top. An infundibulum of maximum diameter > 3 mm would be considered as a remaining CA and concluded into the statistical analysis in our study. We defined a negative case as a patient with no CA regardless of other cerebrovascular diseases. We ensured that all observers understood these definitions, to achieve consistency of interpretation.

Two experienced observers with no knowledge of the clinical history and DSA results interpreted the rotational 4D-TRAK and 3D-TOF-MRA datasets randomly and independently on an offline workstation, using VR with the single artery highlighting approach from multiple viewing angles. MIPs and source images were presented to the observers with appropriate adjustable thresholds of window width and level. When the two observers could not agree on the presence of a CA, a third observer blind to clinical history and DSA results would be invited and the final decision was reached by a majority.

Image quality of 4D-TRAK and 3D-TOF-MRA was assessed on a four-point scale: 1’, inacceptable; 2’, acceptable; 3’, good; and 4’, excellent. The following factors were taken into consideration: background suppression; display of parent artery; display of small terminal branches; venous contamination; and artifacts. Images with a score of 2–4 were considered interpretable. Diagnostic confidence in the presence of a CA on 4D-TRAK and 3D-TOF-MRA was assessed using a previously reported five-point scale: 5, aneurysm definitely absent; 4, aneurysm probably absent; 3, uncertain; 2, aneurysm probably present; and 1, aneurysm definitely present. Cases with one or more aneurysm identified as probably or definitely present were considered positive; all others were considered negative.

### Statistical analysis

The diagnostic performance of 3D-TOF-MRA and 4D-TRAK at 3.0 T were compared with that of 3D-DSA. In a patient-based evaluation, a patient with at least one CA detected by both MRA and DSA was considered as a true-positive (TP) case. A patient with no CA detected by MRA and DSA was considered as a true-negative (TF) case. A patient with at least one CA detected by MRA and no CA detected by DSA was considered as a false-positive (FP) case. A patient with no CA detected by MRA and at least one CA detected by DSA was considered as a false-negative (FN) case. Accuracy, sensitivity, specificity, positive predictive value (PPV) and negative predictive value (NPV) were compared on patient-by-patient level. For patients with multiple CAs, an aneurysm-based evaluation was made as well. Inter-observer reliability was calculated for paired observers of 3D-TOF-MRA and 4D-TRAK using the kappa (κ) statistic. A κ value of 0.8 or above indicated excellent agreement; 0.6–0.8, good agreement; 0.4–0.6, fair agreement; and < 0.4, poor agreement. The statistical analyses were performed using SPSS version 13.0 (SPSS Inc., Chicago, Illinois).

## Results

Fifty-eight CAs were finally found in 52 patients by 3D-DSA, including no CA detected in 7 patients, 1 CA in each of the 34 patients, 2 CAs in each of the 10 patients, and 4 CAs in 1 patient. Nineteen (32.8%) of the 58 aneurysms were < 3 mm in maximal diameter, 25 (43.1%) ≥ 3 mm and < 5 mm, 10 (17.2%) ≥ 5 mm and < 10 mm, and 4 (6.9%) ≥ 10 mm. Nine (15.5%) aneurysms located at the anterior cerebral artery, 6 (10.3%) at the middle cerebral artery, 35 (60.3%) at the internal carotid artery and 8 (13.8%) at the vertebrobasilar system.

The diagnostic accuracy, sensitivity, specificity, PPV and NPV of 4D-TRAK and 3D-TOF-MRA at 3 T for the detection of CA on a patient-based evaluation is described in Table 1. An evaluation based on aneurysm-by-aneurysm level for multiple CAs is described in Table 2 and 3. Subgroup analysis revealed that for 19 very small CAs (maximal diameter <3 mm, measured on 3D-DSA), 9 was missed on 4D-TRAK and 1 on 3D-TOF-MRA (P = 0.008). However, for 39 CAs with maximal diameter ≥ 3 mm, the diagnostic accuracy is equally (39 on 4D-TRAK vs. 39 on 3D-TOF-MRA) (P = 1). The inter-observer reliability of patient-based evaluation, based on the κ statistic for paired observers, was 0.91 and 0.95 on 4D-TRAK and 3D-TOF-MRA in patient-based evaluation, respectively; 0.89 and 0.96 on 4D-TRAK and 3D-TOF-MRA in aneurysm-based evaluation in patients with multiple CAs, respectively.

**Table 1:** Diagnostic performance of 4D-TRAK and 3D-TOF-MRA for the detection of CAs identified on 3D-DSA in the patient-based evaluation

**Technique**	**N**	**TP**	**TN**	**FN**	**k**	**Sensitivity**	**Specify%**	**Specify%**	**PPV%**	**NPV%**	**Accuracy%**
**4D-TRAK**	**52**	**42**	**6**	**1**	**3**	**0.91**	**93.33**	**85.71**	**96.67**	**66.67**	**92.31**
**3D-TOF-MRA**	**52**	**45**	**6**	**1**	**0**	**0.95**	**100.00**	**85.71**	**97.83**	**100**	**98.08**

Note: *N*: number; *TP*: true-positive; *TN*: true-negative; *FP*: false-positive; *FN*: false-negative; κ: kappa statistic; *PPV*: positive predictive value; *NPV*: negative predictive value.

**Table 2:** Diagnostic performance of 4D-TRAK and 3D-TOF-MRA for the detection of multiple CAs identified on 3D-DSA

**Case No./Gender/Age**	**Size (mm)**	**Location**	**4D-TRAK**	**3D-TOF-MRA**
10/F/65	≥ 3	L C7 segment	TP	TP
	≥ 3	Top of Basilar artery	TP	TP
16/F/54	< 3	L C7 segment	FN	TP
	< 3	L C5 segment	TP	TP
	/	R C5 segment	TN	TN
22/M/64	< 3	L M1 segment	FN	TP
	< 3	L C7 segment	TP	TP
26/M/34	≥ 3	L C6 segment	TP	TP
	≥ 3	R C5 segment	TP	TP
30/M/45	< 3	L P4 segment	FN	FN
	< 3	L P2 segment	FN	TP
	< 3	L P3 segment	TP	TP
	/	R C6 segment	TN	TN
	< 3	R M1 segment	FN	TP
31/F/59	/	L C7 segment	FP	TN
	≥ 3	R M1 segment	TP	TP
	≥ 3	R C7 segment	TP	TP
34/M/56	≥ 3	R C4 segment	TP	TP
	≥ 3	L V4 segment	TP	TP
37/F/45	< 3	L Acom	FN	TP
	≥ 3	R C7 segment	TP	TP
43/M/40	< 3	L C6 segment	TP	TP
	< 3	R Acom	TP	TP
45/M/40	≥ 3	L C6 segment	TP	TP
	≥ 3	R C4 segment	TP	TP
52/M/46	< 3	L M1 segment	TP	TP
	≥ 3	R Acom	TP	TP

Note: *M*: male; *F*: female; *L*: left; *R*: right; *Acom*: anterior communicating artery; *TP*: true-positive; *TN*: true-negative; *FP*: false-positive; *FN*: false-negative.

**Table 3:** Diagnostic performance of 4D-TRAK and 3D-TOF-MRA for the detection of CAs identified on 3D-DSA in the aneurysm-based evaluation in patients with multiple CAs

**Technique**	**N**	**TP**	**TN**	**FN**	**k**	**Sensitivity**	**Specify%**	**Specify%**	**PPV%**	**NPV%**	**Accuracy%**
**4D-TRAK**	**19**	**18**	**2**	**1**	**6**	**0.89**	**75.00**	**66.67**	**94.74**	**25.00**	**74.07**
**3D-TOF-MRA**	**23**	**23**	**3**	**0**	**1**	**0.96**	**95.83**	**100.00**	**100.00**	**75.00**	**96.30**

Note: *N*: number; *TP*: true-positive; *TN*: true-negative; *FP*: false-positive; *FN*: false-negative; κ: kappa statistic; *PPV*: positive predictive value; *NPV*: negative predictive value.

**Figure 1:**
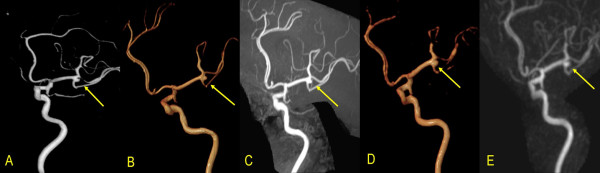
A 50-year-old female patient: A. VR DSA, B. VR 3D-TOF-MRA, C. MIP 3D-TOF-MRA, D. VR 4D-TRAK and E. MIP 4D-TRAK showed a CA located at the bifurcation of the left middle cerebral artery.

**Figure 2:**
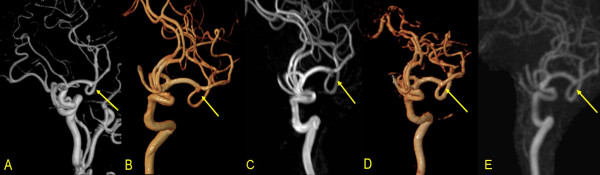
A 45-year-old male patient: A. VR DSA, B. VR 3D-TOF-MRA and C. MIP 3D-TOF-MRA showed a CA located at the bifurcation of left middle cerebral artery. D. VR 4D-TRAK and E. MIP 4D-TRAK missed the aneurysm.

**Figure 3:**
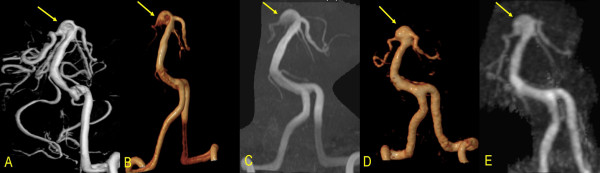
A 65-year-old female patient: A. VR DSA, D. VR 4D-TRAK and E. MIP 4D-TRAK have better characterization of the CA (11 mm in maximum diameter) located at the top of the basal artery than B.VR 3D-TOF-MRA and C. MIP 3D-TOF-MRA.

Summary of misdiagnosed aneurysms on 3D-TOF-MRA and 4D-TRAK is detailed in Table 4. In 52 patients, 58 CAs were visualized on 3D-TOF-MRA, 51 on 4D-TRAK (Figure 1). On 3D-TOF-MRA, 1 patient (Case No.41) without any aneurysm was misdiagnosed due to an acute turn of the siphon at C4. One aneurysm in 1 patient located at left P4 segment (Case No.30) was missed by both the two methods due to its small size and peripheral location. On 4D-TRAK, 2 patients (Case No. 31 and 33) were misdiagnosed as having a aneurysm due to excessively tortuosity at the origin of the posterior communicating artery, and 9 aneurysms were missed in 7 patients (Case No. 3, 16, 21, 22, 27, 30 and 37) with maximal diameter < 3 mm (Figure 2). In 4 patients with a larger aneurysm (≥ 10 mm in diameter), the outline and size can be better ascertained by 4D-TRAK than by 3D-TOF-MRA (Figure 3).

**Table 4:** Summary of false-positive and false-negative aneurysms on 4D-TRAK and 3D-TOF-MRA

**Case No./Gender/Age**	**4D-TRAK**	**3D-TOF-MRA**	**Location**	**Size (mm)**	**Causes**
31/F/59	FP	TN	L C7 segment	≥ 3	Excessively tortuosity at the origin of the Pcom
33/F/58	FP	TN	L C7 segment	≥ 3	
30/M/40	FN	FN	L P4 segment	< 3	Distal location and small size
16/F/54	FN	TP	L C7 segment	< 3	“vascular edge” artifact and spatial resolution compromise of 4D-TRAK technique
37/F/45	FN	TP	L Acom	< 3	
22/M/64	FN	TP	L M1 segment	< 3	
30/M/45	FN	TP	L P2 segment	< 3	
21/M/60	FN	TP	R C4 segment	< 3	
3/M/35	FN	TP	L C5 segment	< 3	
27/M/45	FN	TP	R C5 segment	< 3	
30/M/45	FN	TP	R M1 segment	< 3	
41/M/58	TN	FP	L C4 segment	≥ 3	Acute turn of the siphon at C4 segment

Note: *M*: male; *F*: female; *L*: left; *R*: right; *Acom*: anterior communicating artery; *Pcom*: posterior communicating artery; *TP*: true-positive; *TN*: true-negative; *FP*: false-positive; *FN*: false-negative.

In a semi-quantitative score of image quality of 3D-TOF-MRA images, 40 of 52 cases were regarded as excellent (76.0%), while 10 cases were good (19.2%) and 2 cases were acceptable (3.8%). By contrast, the number of excellent cases on 4D-TRAK was only 21 of 52 (40.4%), with 24 good cases (46.2%) and 7 acceptable cases (13.5%) (P < 0.001). For 6 restless patients with SAH, 4 cases were acceptable, and 2 cases were good on 3D –TOF-MRA (8 min 56 s). On 4D-TRAK (40 s), 2 cases were acceptable, and 4 cases were good.

## Discussion

Time-resolved MRA (TR-MRA) is an alternative MRA technique which uses a keyhole imaging approach to sample the center of k-space more frequently compared with high-spatial-frequency information peripherally to obtain MRA images with high temporal resolution, such as 4D time-resolved MRA with keyhole (4D-TRAK), time-resolved imaging with stochastic trajectories (TWIST), time-resolved echo-shared angiography technique (TREAT), time-resolved imaging of contrast kinetics (TRICKS) [8,10-13]. 4D-TRAK with a combination of parallel imaging and CENTRA at high field strength has demonstrated its wide clinical potential in the characterization of multiple vascular lesions because of the high temporal and spatial resolution obtained simultaneously in previous studies [6-9]. Most ruptured CAs in clinical are ≥ 3 mm in diameter. In our study, the diagnostic accuracy of TRAK for these CAs is equal to that of 3D-TOF-MRA which has been proved to be of excellent sensitivity, accuracy, and correlation with 3D-DSA, as it was demonstrated in the previous studies in a large cohort of patients in our center [14,15]. For uncooperative patients with acute SAH and renal impairment at the same time, both computed tomographic angiography (CTA) and 3D-TOF-MRA could not be considered as the first option because of the renal impairment associated with the iodinated contrast agents and the possibility of more motion artifacts due to a relatively longer acquisition time (8 min 56 s in our study), respectively. Considering the increasing current concern linking high-dose gadolinium (Gd) chelates to NSF in patients with advanced renal failure [16,17], a MRA protocol with low-dose contrast medium was desired for CAs patients with renal impairment. However, the desirable dose is frequently too small to perform both a timing bolus and non time-resolved contrast-enhanced MRA (CE-MRA). Although it was reported that the low-dose contrast medium with the associated shorter duration of bolus may have contributed to a compromise of signal-to-noise ratio (SNR) and contrast-to-noise ratio (CNR) and lead to a suboptimal small-vessel conspicuity [18], benefiting from higher magnetic fields with an associated gain in SNR as well as effects on T1-relaxation of contrast [19,20] and subtraction imaging for background suppression and contrast enhancement [21], the small dose of contrast agent utilized has proved to be sufficient to provide dynamic, functional, as well as morphological information in a wide range of vascular diseases in TR-MRA [22,23]. In our result, 4D-TRAK with a single injection of 10 ml of Gd-DPTA and a relatively shorter acquisition time of 40s was sufficient to provide a frame rate up to 2 seconds and morphological information as well and could become a supplementary method for some uncooperative patients with acute SAH and with renal inadequacy.

However, for small CAs with maximal diameter <3 mm, it does not yet have a diagnostic accuracy comparable with 3D-TOF-MRA. The “vascular edge” artifact (blurring or irregular enhancement of vascular edges) and a compromise of spatial resolution compared with high resolution imaging such as 3D-TOF-MRA may be a diagnostic limitation for the overall image quality of 4D-TRAK and confident evaluation of smaller CAs. The peripheral parts of k-space, necessary for spatial resolution, are acquired with the keyhole reference scan after all dynamics in CENTRA keyhole. The sequence that combined CENTRA keyhole and SENSE in our study was likely to provide images with an increased background noise level and reduced vessel delineation due to absence of gadolinium in wash-out phase compared with CE-MRA. A CA is defined as a saccular protrusion from the side wall or bifurcation of the cerebral arteries without artery emerging at its top. Irregular enhancement of vascular edges would reduce the slope-rates of protrusion on vessel edge, as a result of decreasing the diagnostic tendency of CAs or misdiagnosing as unsmooth arterial walls due to atherosclerosis, especially for CAs with a smaller size.

4D-TRAK could also be potential in depiction the morphology of larger CAs in patients with renal insufficiency. Compared with 3D-TOF-MRA, the decreased sensitivity to saturation and turbulence effect can be the advantage of 4D-TRAK benefiting from its different imaging principle [24,25]. Within larger aneurysms, especially those with partial thrombosis, complex flow is usual found [26]. Similar to Jäge’s results [27], we found 4D-TRAK to characterize the morphology of lesions better than 3D-TOF-MRA compared to 3D-DSA in four larger aneurysms (≥ 10 mm). However, the diagnostic sensitivity of 3D-TOF-MRA was not decreased in these cases.

After referencing the previous studies [28-31], except the decreasing risk of NSF for patients with renal failure, advantages of 4D-TRAK compared with non time-resolved CE-MRA in the detection of CAs include: 1) no need for individual assessment of bolus arrival time; Timing of the Gd bolus relative to acquisition of central k-space is critical for CE- MRA for maximum arterial signal with minimal venous signal [28,29]. It can be performed by a separate timing bolus injection or by bolus-tracking sequences that allow automatic or manual triggering when contrast material is seen in the vessels to be imaged. Compared with CE MRA,no additional bolus timing and time-consuming sequence adjustments are necessary in time-resolved imaging. One simply injects the contrast material and performs the scan simultaneously, because as a result of the high temporal update rate (2 s in our study), one of the volumes will coincide with the arrival of contrast material. For CAs patients accompany with any of the following: pediatric patients with fast contrast media passage, patients with impaired cardiac output, and patients with high dilution volumes, the facilitated application of contrast media without the need for an optimal bolus timing may be especially helpful [30]. 2) less arterial and venous overlap; Although CE-MRA has a high spatial resolution for greater anatomical definition of vessels, there is also an increased chance for arterial and venous overlap, which can limit the diagnostic accuracy of CAs [31]. 3) to have the potential to help the decision-making process of endovascular strategy for CAs because of the ability to follow the course of blood flow with an angiographic overview similar to that of DSA. However, further study is still needed to obtain the higher spatial and temporal resolution for a better mimic of process of angiography.

We acknowledge the following limitations of our study. 1) Our study didn’t obtain conventional non-time resolved CE-MRA sequences at the same time, allowing for avoiding the second contrast injection. 2) Our data were obtained from a relatively small sample size.

## Conclusion

4D-TRAK at a lower contrast dose of 10 ml with a combination of SENSE and CENTRA at 3 T could provide similar diagnostic accuracy rate for CAs with maximal diameter ≥ 3 mm, and a better characterization of morphology for larger CAs with maximal diameter ≥ 10 mm compared to 3D-TOF-MRA. However, further study is still needed to improve the “vascular edge” artifact and the compromise in spatial resolution in depiction of CAs with maximal diameter< 3 mm.

## Competing interests

Both authors declare that they have no competing interests.

## Authors’ contributions

WQ and LMH both carried out the study design, patients selection and inclusion, image analysis, statistical analysis and drafted the manuscript. WQ and LMH made the revision and approved the final manuscript. All authors read and approved the final manuscript.

## Pre-publication history

The pre-publication history for this paper can be accessed here:

http://www.biomedcentral.com/1471-2377/12/50/prepub
